# Prevalence and associated factors of dysphonia in non-hospitalized Thai COVID-19 patients: a descriptive study with Thai-VHI10 Assessment

**DOI:** 10.2478/abm-2024-0037

**Published:** 2024-12-16

**Authors:** Thitaree Suwannutsiri, Peerada Arreenich, Premsuda Sombuntham

**Affiliations:** Taksin Hospital, Medical Service Department, Bangkok Metropolitan Administration, Bangkok 10600, Thailand; Department of Otolaryngology-Head and Neck Surgery, King Chulalongkorn Memorial Hospital, Bangkok 10330 Thailand; Department of Otolaryngology-Head and Neck Surgery, Faculty of Medicine, Chulalongkorn University, Bangkok 10330, Thailand

**Keywords:** COVID-19, COVID-19 vaccines, dysphonia, respiratory tract infection, VHI-10, voice disorder

## Abstract

**Background:**

The COVID-19 pandemic first emerged in December 2019 and rapidly spread globally, including Thailand. While respiratory symptoms remain the primary manifestation of the disease, upper respiratory tract symptoms, including dysphonia, have been reported in various studies.

**Objectives:**

To determine the prevalence of dysphonia in non-hospitalized Thai COVID-19 patients and identify associated factors using the Thai-Voice Handicap Index-10.

**Methods:**

This study investigates the prevalence of dysphonia and associated factors in non-hospitalized Thai COVID-19 patients. Conducted from September 2022 to February 2023, it enrolled healthcare workers who tested positive for COVID-19 but were not hospitalized.

**Results:**

Among 82 patients, 53 (64.6%) reported dysphonia, which was significantly associated with cough (*P* = 0.013) and nasal discharge (*P* = 0.047). Substantial improvement was observed at the 3-month follow-up (73.6%). Vaccination may serve as a protective factor (crude odds ratio < 1).

**Conclusion:**

The prevalence of dysphonia among non-hospitalized COVID-19 patients in Thailand is 63.6%, linked to cough and nasal congestion, with symptoms likely to subside within 3 months.

The COVID-19 pandemic, stemming from the novel coronavirus SARS-CoV-2, was first reported in December 2019 in Wuhan, Hubei Province, China [1]. This virulent contagion rapidly disseminated across the globe, including Thailand, where initial outbreaks were identified in January 2020.

Initially, it was discovered that 80% of COVID-19 patients exhibited mild or no respiratory symptoms, while 15%–20% experienced severe pneumonia. Subsequently, mutations in the 2019 coronavirus emerged, altering the severity of the illness according to each strain. Nevertheless, respiratory symptoms remain the principal manifestations of the infection. Symptoms involving the upper respiratory tract are also indicative of the coronavirus infection, including sore throat, cough, nasal congestion, runny nose, anosmia, dysgeusia, phlegm production, and wheezing [2, 3].

Previous studies reported dysphonia in 26.8% [4], 43.7% [5] of COVID-19 patients in Europe and 22.3% [6] of COVID-19 patients in Iraq. It was associated with cough, rhinitis, and dyspnea [5] and mostly recovered within 3 months [7].

The primary objective of this study was to determine the prevalence of dysphonia in non-hospitalized Thai COVID-19 patients using the Thai-Voice Handicap Index-10 (VHI-10). The secondary objective was to identify factors associated with dysphonia in COVID-19 patients.

## Materials and methods

This descriptive study collected data from September 2022 to February 2023, after receiving approval from the institutional review board of the Faculty of Medicine, Chulalongkorn University (IRB No. 653/65). Participants were selected from healthcare workers who had tested positive for COVID-19 using real-time polymerase chain reaction (RT-PCR) and/or antigen test kits (ATK) within 3 months prior to enrollment in the study at King Chulalongkorn Memorial Hospital, Thai Red Cross Society.

Eligible individuals infected with COVID-19 were thoroughly informed about the research by the investigator and subsequently provided with an informed consent form. They meticulously filled out a case record form and evaluated their voice disorders utilizing the Thai-VHI-10 questionnaire, considering their vocal state during the COVID-19 infection. Three months after recovering from COVID-19, participants repeated the VHI-10 questionnaire assessment.

The inclusion criteria for study participants were as follows: (1) aged between 18 years and 65 years old and (2) positive PCR or ATK results within 3 months prior to participating in the research. The exclusion criteria for study participants/volunteers were as follows: (1) severe COVID-19 symptoms or requiring intubation for breathing assistance; (2) pre-existing vocal abnormalities before COVID-19 infection; (3) prior history of head, neck, nose, and sinus surgeries; (4) use of inhaled corticosteroids; and (5) inability to understand or communicate in Thai.

Statistical analysis was performed using IBM SPSS Statistics for Windows, Version 25.0 (IBM Corp., Armonk, NY, USA, 2015). Descriptive statistics were used to analyze demographic data and clinical characteristics of the participants, while the Wilcoxon signed-rank test was utilized to compare pre- and post-COVID-19 VHI-10 scores. Crude and adjusted odds ratios (ORs) with their respective 95% confidence intervals (CI) were computed to determine univariate and multivariate associated factors of dysphonia after COVID-19 infection. The significance level for all statistical tests was set at *P* < 0.05.

## Results

Eighty-two individuals who tested positive for the novel coronavirus participated in our study, with a female majority constituting 84.1% of the cohort, while 15.9% were male. The participants' ages ranged from 22 years to 60 years, with a mean age of 34.67 years. A substantial portion of the subjects, 43.9%, had a body mass index (BMI) within the range of 18.5–22.9 kg/m^2^, followed by those with a BMI of 23.0–29.9 kg/m^2^ (29.3%), those with a BMI <18.5 kg/m^2^ (15.9%), and those with a BMI of 30.0 kg/m^2^ or greater (11%). The most prevalent BMI range was 18.5–22.9 kg/m^2^, accounting for 43.9% of participants. Three individuals had underlying health conditions, including two with persistent respiratory illness (2.4%) and one with cerebrovascular disease (1.2%). The prevalence of smokers was 3.7%, while 20.7% consumed alcoholic beverages, and 4.9% reported experiencing heartburn. The demographic data of the participants are shown in **Table 1**.

**Table 1.** General characteristics of the participants

	**N = 82**
Sex, n (%)	
Male	13 (15.9)
Female	69 (84.1)
Age (years), mean ± SD	34.67 ± 9.76
BMI level (kg/m^2^), n (%)	
<18.5	13 (15.9)
18.5–22.9	36 (43.9)
23.0–29.9	24 (29.3)
>30.0	9 (11.0)
Underlying disease, n (%)	3 (3.7)
Chronic respiratory disease	2 (2.4)
Stroke	1 (1.2)
Smoking, n (%)	3 (3.7)
Alcohol consumption, n (%)	17 (20.7)
Heartburn, n (%)	4 (4.9)
Symptoms of COVID-19, n (%)	
Cough	73 (89.0)
Nasal discharge	64 (78.0)
Sputum	68 (82.9)

BMI, body mass index; SD, standard deviation.

The analysis of the VHI-10 disclosed a marked improvement in voice handicap scores from COVID-19 infection to 3 months post-recovery. The median VHI-10 scores at the time of COVID-19 infection and 3 months later were 11.5 (interquartile range [IQR] 2.75, 21) and 0 (IQR 0, 3), respectively, and this reduction was determined to be statistically significant (*P* < 0.001). The incidence of voice abnormalities during COVID-19 infection and 3 months post-recovery was 64.6% (95% CI = 53.3, 74.9) and 17.1% (95% CI = 9.7, 27.0), respectively. Patients who had voice abnormalities at the time of COVID-19 infection (VHI-10 scores >7) showed substantial improvement at the 3-month follow-up, with a prevalence of 73.6%. However, a significant proportion of patients (26.4%, 95% CI = 15.3, 40.3) still experienced voice abnormalities 3 months post-recovery, as reflected in **Table 2**.

**Table 2.** Voice Handicap Index-10

	**During COVID-19 infection**	**3 months after COVID-19 infection**	***P***
VHI-10, median (IQR)	11.5 (2.75, 21)	0 (0, 3)	<0.001^*^
VHI-10 (n = 82), n (%)			
<7 (Normal)	29 (35.4)	68 (82.9)	<0.001^*^
>7 (Dysphonia)	53 (64.6)	14 (17.1)	
VHI-10 improve (n = 53), n (%)			
<7 (Normal)		39 (73.6)	
>7 (Dysphonia)		14 (26.4)	

Data were analyzed using the Wilcoxon signed-rank test and the McNemar test.

* Statistically significant at the 0.05 level.

IQR, interquartile range; VHI-10, Voice Handicap Index-10.

**Table 3** displays the univariate analysis of the study variables associated with vocal abnormalities in COVID-19 patients. It was found that the symptom of cough during COVID-19 infection was significantly associated with vocal abnormalities in COVID-19 patients at a statistical level of 0.05 (crude OR 8.11 [95% CI = 1.56, 42.21]; *P* = 0.013). The symptom of nasal discharge during COVID-19 infection was also significantly associated with vocal abnormalities in COVID-19 patients at a statistical level of 0.05 (crude OR 2.96 [95% CI = 1.01, 8.66]; *P* = 0.047). Although the number of booster doses of the mRNA vaccine (1, 2, or 3) did not show a significant association with vocal abnormalities in COVID-19 patients (*P* > 0.05), considering only the crude OR values, it was found that the vaccine may be a protective factor against vocal abnormalities in COVID-19 patients, with OR values <1.00.

**Table 3.** Univariate analysis of factors related to dysphonia following COVID-19 infection

**Factors**	**Dysphonia (n = 53)**	**Normal (n = 29)**	**Crude OR (95% CI)**	***P***
BMI level (kg/m^2^), n (%)				
18.5–22.9	25 (47.2)	11 (37.9)	Reference	
<18.5	6 (11.3)	7 (24.1)	0.38 (0.10, 1.39)	0.142
23.0–29.9	16 (30.2)	8 (27.6)	0.88 (0.29, 2.66)	0.821
>30.0	6 (11.3)	3 (10.3)	0.88 (0.19, 4.17)	0.872
Smoking, n (%)	2 (3.8)	1 (3.4)	1.10 (0.09, 12.65)	0.940
Alcohol consumption, n (%)	13 (24.5)	4 (13.8)	2.03 (0.60, 6.93)	0.258
Heartburn, n (%)	4 (7.5)	0 (0.0)	NA	
Symptoms of COVID-19, n (%)				
Cough	51 (96.2)	22 (75.9)	8.11 (1.56, 42.21)	0.013^*^
Nasal discharge	45 (84.9)	19 (65.5)	2.96 (1.01, 8.66)	0.047^*^
Sputum	47 (88.7)	21 (72.4)	2.98 (0.92, 9.68)	0.069
Number of COVID-19 symptoms, n (%)				
None/1 symptom	2 (3.8)	9 (31.0)	Reference	
2 Symptoms	12 (22.6)	6 (20.7)	9.00 (1.46, 55.48)	0.018^*^
3 Symptoms	39 (73.6)	14 (48.3)	12.54 (2.41, 65.23)	0.003^*^
Number of booster dose with mRNA vaccine, n (%)				
1 dose	8 (15.8)	4 (13.9)	0.50 (0.04, 6.08)	0.500
2 doses	22 (43.1)	13 (44.8)	0.42 (0.04, 4.20)	0.423
3 doses	17 (33.3)	11 (37.9)	0.39 (0.04, 3.93)	0.386
None	4 (7.8)	1 (3.4)	Reference	

NA: *n* = 0 in crosstab table. Data were analyzed using simple logistic regression

* Statistically significant at the 0.05 level.

BMI, body mass index; CI, confidence interval; OR, odds ratio.

**Table 4** shows the analysis of BMI as a factor, symptoms during COVID-19, and receiving the mRNA booster vaccine in relation to abnormal voice in COVID-19 patients. The results showed that symptoms of fever during COVID-19 were significantly associated with abnormal voice (adjusted OR 8.53 [95% CI = 1.41, 51.45]; *P* = 0.019). This means that patients with abnormal voice had 8.53 times higher odds of having a fever as a symptom of COVID-19 compared to those without abnormal voice. Meanwhile, BMI, symptoms of runny nose and throat mucus, and receiving the mRNA booster vaccine were not statistically related to abnormal voice in COVID-19 patients (*P* > 0.05).

**Table 4.** Multivariate analysis of factors related to dysphonia following COVID-19 infection

**Factors**	**Adjusted OR (95% CI)**	***P***
BMI level (kg/m^2^)		
18.5–22.9	Reference	
<18.5	0.30 (0.07, 1.33)	0.113
23.0–29.9	0.49 (0.14, 1.74)	0.268
>30.0	0.76 (0.13, 4.58)	0.762
Symptoms of COVID-19		
Cough	8.53 (1.41, 51.45)	0.019^*^
Nasal discharge	1.99 (0.56, 7.02)	0.287
Sputum	2.50 (0.61, 10.17)	0.201
At least one dose of mRNA vaccine	0.68 (0.20, 2.28)	0.531

Data were analyzed using multiple logistic regression (Enter method).

* Statistically significant at the 0.05 level.

BMI, body mass index; CI, confidence interval; OR, odd ratio.

## Discussion

The human voice originates from air produced by the lungs, which passes through the vocal cords and is articulated into speech as it traverses the larynx and mouth. When infected with the 2019 novel coronavirus, abnormalities can arise from the lungs, which serve as the genesis of the air responsible for vocalization. Additionally, symptoms such as coughing, phlegm, and nasal discharge can have a detrimental impact on vocal quality [3,4,5].

The assessment of abnormal vocal conditions involves various methods, with the VHI questionnaire being a widely used and globally translated tool. VHI evaluates vocal abnormalities by having patients respond to questions that assess the impact of their voice on their quality of life. It can be used as part of the diagnostic process for abnormal vocal conditions, as previous studies have demonstrated the questionnaire's sensitivity in detecting voice abnormalities. The original version consists of 30 questions, but the more prevalent version is the VHI-10, which comprises 10 selected questions from the original questionnaire. A study by Clark et al. [8] assessed the accuracy of the VHI-10 and found it to be comparable to the VHI-30, despite having fewer questions. The VHI-30 questionnaire was translated and validated into Thai by Jaruchinda et al. [9]. Subsequently, the Thai version of the VHI-30 was further validated and condensed into the VHI-10 questionnaire [10]. In our study, we used the validated Thai version of the VHI-10 as a voice assessment tool for participants with COVID-19 infection.

Our study, which was conducted during the Omicron outbreak period in Thailand between September and December 2022 [11], found that the prevalence of dysphonia among non-hospitalized COVID-19 patients was 63.6%, surpassing the prevalence reported in previous studies from 2020 to 2021. This disparity is likely attributable to the variance in COVID-19 strains, which can result in differing symptoms. Notably, the majority of subjects in our study were females, as it is well established that females are more susceptible to voice disorders than males [12].

However, the symptoms related to voice abnormalities were reported as cough and concomitant nasal discharge, which were in accordance with prior studies [4,5,6]. Studies by Al-Ani et al. [7] and Verma et al. [13] have established that the majority of patients with voice abnormalities experience persistent symptoms for over a month and improvement after 3 months. This study also found that 73.6% of patients showed improvement after 3 months and that the second VHI-10 score was statistically significantly lower.

Other factors associated with dysphonia include BMI, smoking, alcohol consumption, and heartburn [14, 15], yet this study found no statistically significant relationships. The presence of heartburn, indicating acid reflux, was only observed in the group with voice abnormalities. The researchers attempted to control for confounding variables, such as pre-existing respiratory issues, prior head and neck surgeries, and topical steroid use, to increase the reliability of the data.

The multifaceted impact of COVID-19 on vocal health draws attention to intriguing intersections between infection, treatment, and resultant dysphonia. Though primarily a respiratory ailment, COVID-19 demonstrates a substantive impact on voice quality, evidenced by a prevalence of dysphonia in patients, as per the referenced studies [5]. Dysphonia in the context of COVID-19 can emanate from direct and indirect pathways. Direct implications involve the viral impact on laryngeal tissues, where the infection could induce inflammation and, consequently, voice disturbances. Alternatively, Lechien et al. [16] and Vance et al. [17] have pondered whether dysphonia emerges indirectly, potentially consequent to the treatment regimens, such as the employment of inhaled steroids, which have a known propensity to affect voice quality by inducing laryngeal myopathies or mucosal dryness. The patient's general health, vocal load, and previous vocal health must be recognized as contributory factors. Furthermore, an investigation into the rehabilitative journey of the voice, especially in professional voice users like singers [17], becomes pivotal, thereby crafting a comprehensive exploration of COVID-19's implications on vocal health and outlining an imperative trajectory for future research and clinical practice. This discussion urges the necessity to delineate between dysphonia emerging as a direct symptom of COVID-19 or as a byproduct of its treatment modalities, emphasizing an integrated approach to voice care within the context of the pandemic.

The efficacy of mRNA vaccines as a booster dose has been supported by several studies [18,19,20]. The study revealed that the Omicron strain is associated with fewer instances of loss of smell and taste but has a higher incidence of upper respiratory tract symptoms, including hoarseness. However, there was no statistically significant difference in symptomatology between individuals who received the primary series vaccine and those who received the primary series plus booster [21]. Our study thus sought to examine the relationship between mRNA vaccination and dysphonia, despite the lack of statistical correlation. Upon considering crude OR values, it was found that the value was <1.00, suggesting that mRNA vaccination may serve as a protective factor against voice abnormalities in COVID-19 patients.

In this study, there were limitations, including the examination of the study population being primarily female healthcare workers who were healthy and the use of subjective assessments without objective evaluations. Additionally, the sample size was not particularly large.

## Conclusion

The prevalence of voice disorders, or dysphonia, among non-hospitalized individuals diagnosed with COVID-19 in Thailand was reported to be 63.6%. This affliction was linked to coughing and nasal congestion, but the study suggests that the symptoms of dysphonia were likely to subside within 3 months. However, it should be noted that the study has limitations, including reliance on subjective evaluations and a limited sample size, which may affect the accuracy of these findings. Further investigations are encouraged, including more comprehensive and objective evaluations and expanding the sample population to include a more diverse range of individuals.
